# Supplementary material for: The interactome of intact mitochondria by cross-linking mass spectrometry provides evidence for coexisting respiratory supercomplexes
Source: Mol Cell Proteomics. 2017 Dec 8;17(2):216–32. doi: 10.1074/mcp.RA117.000470 (PMC5795388; doi:10.1074/mcp.RA117.000470)
Supplement: Supplemental Data [file supp_RA117.000470_133922_0_supp_23978_fzffwf.zip › spectra_annotation/mito_DR_spectra_annotation/162-1-13-1-10-1.pdf]

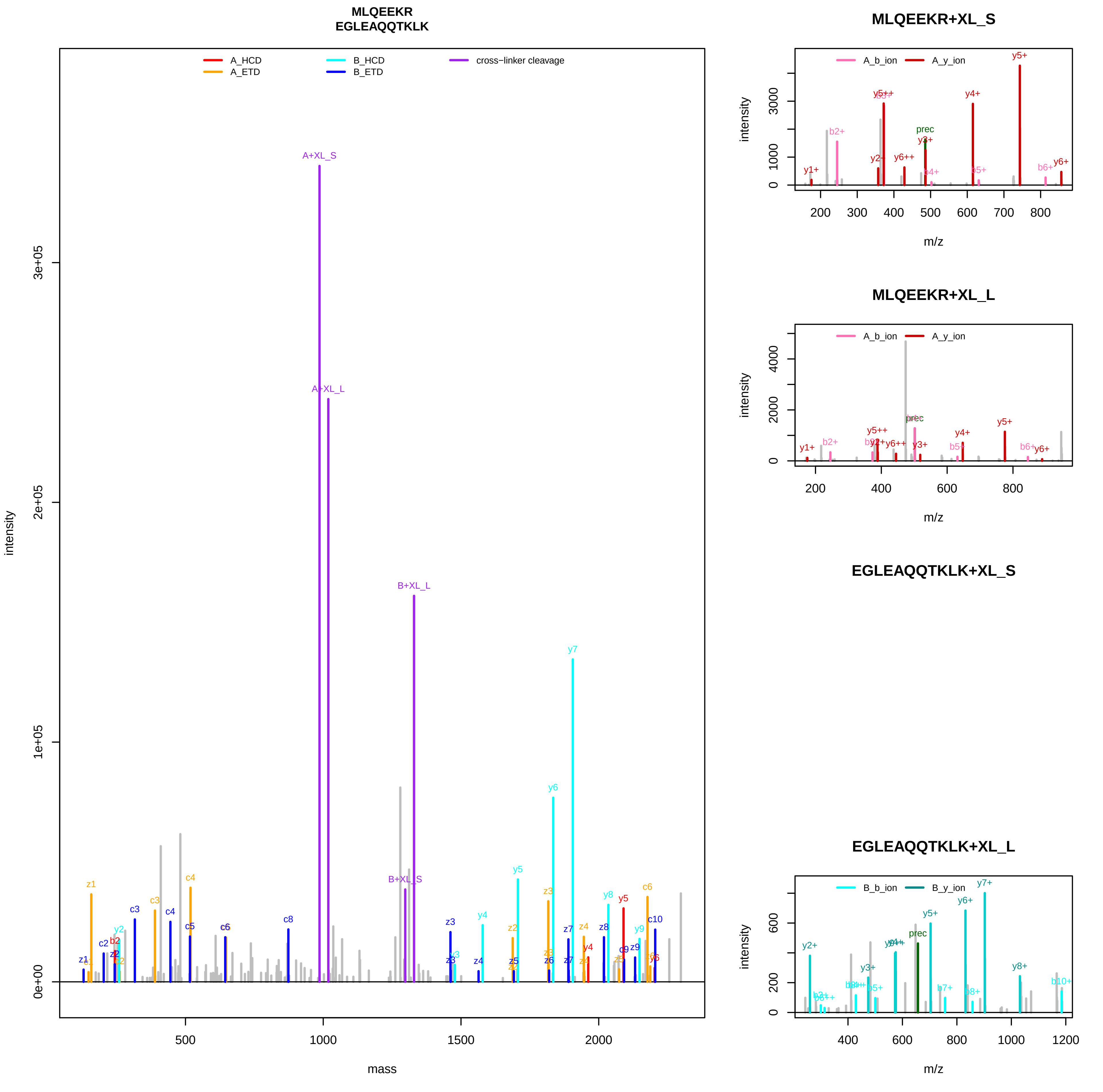

MLQEEKR
EGLEAQQTKLK
A_HCD
A_ETD
B_HCD
B_ETD
cross−linker cleavage
A+XL_S
A+XL_L
B+XL_L
B+XL_S
intensity
mass
0e+00
1e+05
2e+05
3e+05
500
1000
1500
2000
z1
c1
c2
b2
z2
y2
c3
c3
c4
c4
c5
c6
c8
z3
y3
z4
y4
z2
z5
y5
z3
z6
y6
z7
y7
z4
y4
z8
y8
y5
z9
c9
y9
c10
c6
y6
MLQEEKR+XL_S
A_b_ion
A_y_ion
m/z
y1+
b2+
y2+
y5++
y6++
prec
y3+
b4+
y4+
b5+
y5+
b6+
y6+
MLQEEKR+XL_L
EGLEAQQTKLK+XL_S
EGLEAQQTKLK+XL_L
B_b_ion
B_y_ion
y2+
y3+
y4+
y5+
y6+
y7+
y8+
b7+
b8+
b10+
